# Implications in the difference of anti-Mi-2 and -p155/140 autoantibody prevalence in two dermatomyositis cohorts from Mexico City and Guadalajara

**DOI:** 10.1186/ar4207

**Published:** 2013-04-04

**Authors:** Marcelo H Petri, Minoru Satoh, Beatriz T Martin-Marquez, Raul Vargas-Ramírez, Luis J Jara, Miguel A Saavedra, Claudia Cruz-Gonzalez, Lilia Andrade-Ortega, Olga Vera-Lastra, Mario Salazar-Páramo, Rosa E Prieto-Parra, Laura Gonzalez-Lopez, Jorge I Gamez-Nava, Hermes U Ramírez-Sánchez, Jason YF Chan, Steven J Ross, Edward KL Chan, Mónica Vázquez-Del Mercado

**Affiliations:** 1 Departamento de Biología Molecular y Genómica, Instituto de Investigación en Reumatología y del Sistema Músculo Esquelético (IIRSME), Sierra Mojada 950, Planta baja, Edificio P Ala oriente, Centro Universitario de Ciencias de la Salud, Universidad de Guadalajara, Guadalajara, Jalisco, CP 44340 Mexico; 2 Division of Rheumatology and Clinical Immunology, Department of Medicine, University of Florida, 1600 SW Archer Rd, Gainesville, FL 32610-0221, USA; 3 Department of Pathology, Immunology, and Laboratory Medicine, University of Florida, 1600 SW Archer Rd, Gainesville, FL 32610-0221, USA; 4 Direction of Education and Research, Hospital de Especialidades Centro Médico La Raza, IMSS, Calzada Vallejo y Jacarandas S/N, Mexico City, CP 02990, Mexico; 5 Servicio de Reumatología, Hospital La Raza, IMSS, Cd. de México, Calzada Vallejo y Jacarandas S/N, CP 02990, Mexico; 6 Centro Médico Nacional 20 de Noviembre, ISSSTE, Felix Cuevas 540 Del Valle 03100, D.F., México; 7 División de Medicina Interna, Hospital La Raza, IMSS, Cd de México, Calzada Vallejo y Jacarandas S/N, CP 02990, Mexico; 8 División de Investigación, CMNO, IMSS, Departamento de Fisiología del Centro Universitario de Ciencias de la Salud, Universidad de Guadalajara, Av. Belisario Domínguez 1000, SL. Guadalajara, JL 44349, Mexico; 9 Servicio de Reumatología, CMNO, IMSS, Av. Belisario Dominguez 1000, Guadalajara, JL 44349, Mexico; 10 Servicio de Medicina Interna Reumatología, Hospital General Regional 110, IMSS, Prolongacion Circunvalacion Oblatos 2208 Guadalajara, Jalisco, CP 44700, Mexico; 11 UMAE, CMNO, IMSS, Guadalajara, Guadalajara, Jalisco, Av. Belisario Dominguez 1000, CP 44349, Mexico; 12 Instituto de astronomia y Meteorologia, Departamento de Fisica. Division de Ciencias Basicas, Universidad de Guadalajara, Av. Vallarta 2602, CP44130, Mexico; 13 Department of Oral Biology, University of Florida, Gainesville, FL 32610-0221, USA; 14 División de Medicina Interna, Servicio de Reumatología, Hospital Civil Dr. Juan I. Menchaca, Guadalajara, Jalisco, Salvador Quevedo y Zubieta S/N, CP 44240, Mexico

## Abstract

**Introduction:**

Autoantibodies and clinical manifestations in polymyositis/dermatomyositis (PM/DM) are affected by both genetic and environmental factors. The high prevalence of DM and anti-Mi-2 in Central America is thought to be associated with the high UV index of the area. The prevalences of autoantibodies and the clinical manifestations of PM/DM were evaluated comparing two cohorts in Mexico.

**Methods:**

Ninety-five Mexican patients with PM/DM (66 DM, 29 PM; 67 Mexico City, 28 Guadalajara) were studied. Autoantibodies were characterized by immunoprecipitation using ^35^S-methionine labeled K562 cell extract. Clinical information was obtained from medical records.

**Results:**

DM represented 69% of PM/DM and anti-Mi-2 was the most common autoantibody (35%), followed by anti-p155/140 (11%); however, anti-Jo-1 was only 4%. The autoantibody profile in adult-onset DM in Mexico City versus Guadalajara showed striking differences: anti-Mi-2 was 59% versus 12% (*P *= 0.0012) whereas anti-p155/140 was 9% versus 35% (*P *= 0.02), respectively. A strong association of anti-Mi-2 with DM was confirmed and when clinical features of anti-Mi-2 (+) DM (*n *= 30) versus anti-Mi-2 (-) DM (*n *= 36) were compared, the shawl sign (86% versus 64%, *P *< 0.05) was more common in the anti-Mi-2 (+) group (*P *= 0.0001). Levels of creatine phosphokinase (CPK) were higher in those who were anti-Mi-2 (+) but they responded well to therapy.

**Conclusions:**

Anti-Mi-2 has a high prevalence in Mexican DM and is associated with the shawl sign and high CPK. The prevalence of anti-Mi-2 and anti-p155/140 was significantly different in Mexico City versus Guadalajara, which have a similar UV index. This suggests roles of factors other than UV in anti-Mi-2 antibody production.

## Introduction

Autoantibodies in polymyositis/dermatomyositis (PM/DM) are clinically useful biomarkers. Anti-Jo-1 antibodies that recognize histidyl-tRNA synthetase is a well established serological biomarker for PM/DM [1,2] known for more than 30 years and commercial tests have been widely available to clinicians [3]. There are many other autoantibodies specific for a diagnosis of PM/DM (myositis-specific autoantibodies (MSAs)) and that are also associated with unique subsets of the disease and help in predicting organ involvement, treatment outcome and prognosis [1,2]; however, their clinical usage is limited because their standard screening test is radioimmunoprecipitation, which has been performed only at a limited number of institutions in US, UK and a few other European countries, and Japan. Thus, information on the prevalence and clinical association of other MSAs is based on data from limited sources because data on MSAs in other countries are scarce. Nevertheless, based on available information, the prevalence of MSAs appears to be quite different in different countries [4-8] or even within the same country [9-11], suggesting an interesting interaction of genetic and environmental factors in the production of MSAs. In particular, a few previous studies [5,6] reported an increased percentage of DM and prevalence of anti-Mi-2 antibodies in PM/DM patients in Central America and suggested a role of UV radiation in the development of DM and anti-Mi-2 antibodies. We aimed at determining the prevalence and clinical association of MSAs in two Mexican cohorts with PM/DM, focusing on anti-Mi-2 autoantibodies.

## Methods

### Patients

Ninety-five consecutive patients with PM/DM (29 PM, 66 DM) who visited adult rheumatology clinics in 2009 to 2012 and were selected based on Bohan's criteria [12] were enrolled in the study. Five juvenile-onset DM (JDM) cases from the same clinics were also enrolled. Twenty-eight cases (8 PM, 20 DM including 3 JDM) were from Guadalajara (Hospital Civil Dr. Juan I. Menchaca, Hospital General Regional 110, IMSS, UMAE, CMNO, IMSS) and 67 cases (21 PM, 46 DM including 2 JDM) were from Mexico City (Hospital La Raza, IMSS, Hospital 20 de Noviembre, ISSSTE). Clinical information was obtained from medical records. The protocol was approved by the Institutional Review Board (IRB) of the Centro Universitario de Ciencias de la Salud, Universidad de Guadalajara and by the Hospital Civil de Guadalajara Dr. Juan I. Menchaca under the register 969/10. This study meets and is in compliance with all ethical standards in medicine, and informed consent was obtained from all patients according to the Declaration of Helsinki.

### Determination of autoantibodies

Autoantibodies in sera were screened by immunoprecipitation (IP) using ^35^S-methionine labeled K562 cell extracts [13]. Specificity of the autoantibodies was determined using previously described reference sera. Analysis of RNA components of the autoantigens was by urea-PAGE and silver staining (Silver Stain Plus, Bio-Rad, Hercules, CA, USA) [10]. Recombinant Ro52 protein was expressed and purified as described [14] and Jo-1 was purchased from Abazyme (Needham, MA, USA). Anti-Ro52 and -Jo-1 antibodies were tested by ELISA using these recombinant proteins at 1:500 serum dilution as described [8]. Optical density (OD) of the samples was converted into units using a standard curve created by a prototype positive serum [15].

### Immunofluorescent antinuclear antibodies

Immunofluorescent antinuclear/cytoplasmic antibodies (HEp-2 ANA slides; INOVA Diagnostics, San Diego, CA, USA) were tested using a 1:80-diluted human sera followed by DyLight 488 donkey immunoglobulin G (IgG) F(ab)'2 anti-human IgG secondary antibodies (1:200 dilution, γ-chain-specific, Jackson ImmunoResearch Laboratories, Inc. West Grove, PA, USA).

### Statistical analysis

All parameters were analyzed by the Mann-Whitney test, Wilcoxon matched-pairs signed ranked test or Fisher's exact test between groups using Prism 6.0b for Macintosh (GraphPad Software, Inc. La Jolla, CA, USA). Statistical significance was accepted at *P *< 0.05. When statistical significance was observed in comparing the prevalence of autoantibodies, 95% CI also was calculated.

## Results

**Figure 1:**
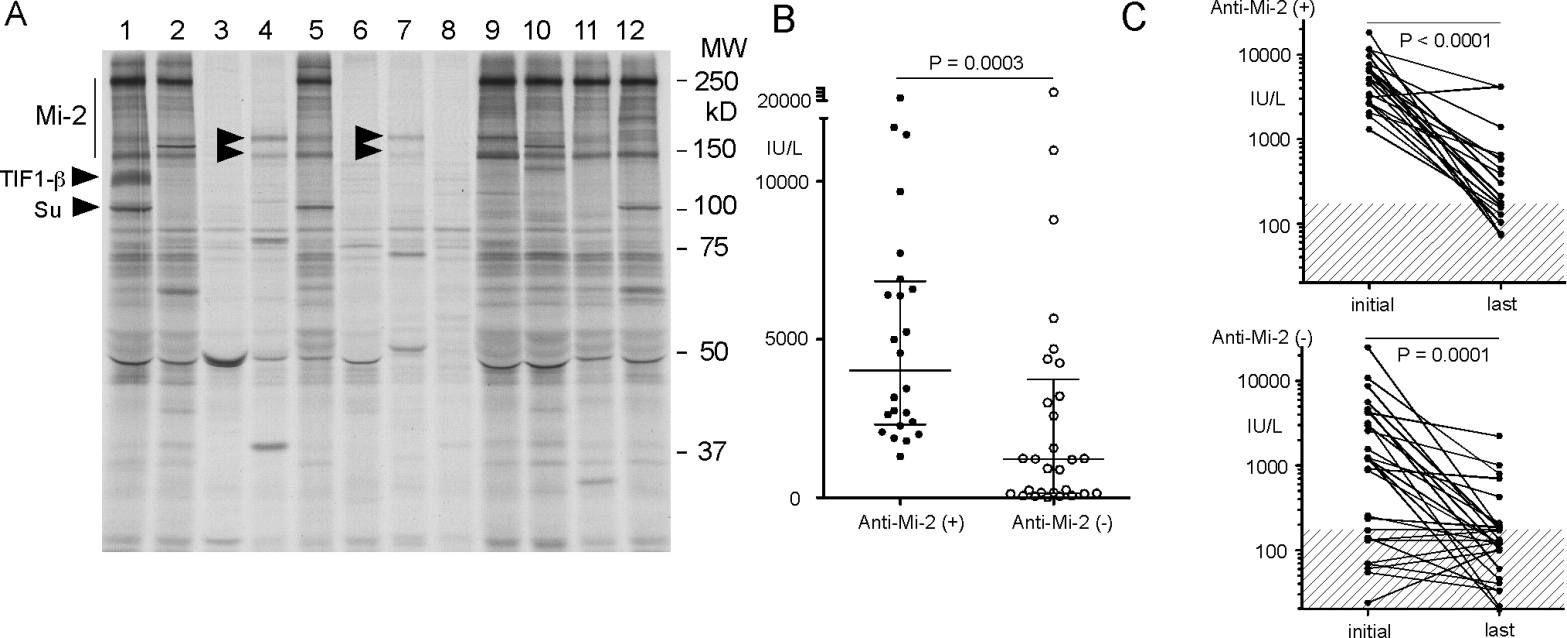
**Autoantibodies by immunoprecipitation and CPK levels in Mexican patients with PM/DM**. **A**. Immunoprecipitation using sera from Mexican patients with PM/DM. ^35^S-methionine labeled K562 cell extract was immunoprecipitated using sera from PM/DM patients. Molecular weight markers and positions of Mi-2, TIF-1β, Su and p155/140 (arrowheads) are indicated. **B**. CPK levels at initial visit are significantly higher in anti-Mi-2 (+) than anti-Mi-2 (-) DM patients. CPK levels of DM patients at initial visit are plotted. Lines in each group indicate median + interquartile range. *P *= 0.0003 by Mann-Whitney. **C**. CPK levels at initial and last visit in each DM patient compared between anti-Mi-2 (+) and (-) groups. Top, anti-Mi-2 (+) group, initial versus last visit, *P *< 0.0001 by Wilcoxon matched pairs test. CPK, creatine phosphokinase; PM/DM, polymyositis/dermatomyositis.

DM was dominant in Mexican patients with PM/DM representing 69% (66/95), consistent with the high percentage of DM in Mexico reported in the literature [6]. The percentage of DM in Mexico City and Guadalajara was similar; 69% (46/67) and 71% (20/28), respectively. During screening of the serum samples by IP, it was apparent that anti-Mi-2 [16] and -p155/140 (TIF-1γ/α) [17,18] were the two main autoantibody specificities in this cohort (Figure 1A). Anti-Jo-1 which is the most common MSA in the majority of studies from other countries [4,19], was only 4% while anti-Mi-2 was 35% and anti-p155/140 was 11% (Table 1). Other MSA specificities listed were a few percent each and antibodies to aminoacyl tRNA synthetases (ARS) other than Jo-1, such as PL-7, PL-12, EJ and OJ, were not found. As in all previous reports in the literature, anti-Mi-2 (45% in DM versus 10% in PM, *P *< 0.0001) and - p155/140 (15% versus 0%, *P *< 0.05) were mainly found in DM (Table 1). Anti-MJ and SAE (small ubiquitin-like modifier activating enzyme) that are also associated with DM [8,20] were found in 5% and 3%, respectively, of DM patients but not in PM patients. Patients negative for MSA were 21% in DM and 69% in PM cohorts (*P *< 0.0001). Other autoantibodies not specific for PM/DM, such as anti-Su/Ago2 [21], Ro60 and Ro52, were found in both PM and DM. DM patients were classified into adult-onset DM and JDM (Table 1) and only adult-onset DM patients were further analyzed comparing patients from Mexico City versus Guadalajara (Table 2). Adult-onset DM patients from Guadalajara were slightly younger than those from Mexico City and striking differences in the prevalence of autoantibodies between the two cohorts were noted; the prevalence of anti-Mi-2 was 59% (95% CI, 43.3 to 73.7) in the Mexico City cohort but only 12% (95% CI, 1.5 to 36.4) in the Guadalajara cohort (*P *= 0.0012 by the Fisher exact test, Table 2). In contrast, the prevalence of anti-p155/140 showed an opposite trend in which the prevalence was 9% (95% CI, 2.5 to 21.7) in Mexico City DM patients versus 35% (95% CI, 14.2 to 61.7) in Guadalajara DM patients (*P *= 0.02). No other significant differences in prevalence of MSA were noted between the two cohorts. Since the majority of anti-Mi-2 positive patients had DM (30 DM, 3 PM), the clinical features of the anti-Mi-2 positive patients were compared with those of the anti-Mi-2 negative patients, only in DM patients (Table 3, left). Since the number of JDM is small (two from Mexico City and three from Guadalajara), and most of them were close to age 18 and some were already older than age 18, JDM cases were included in this analysis. One each from the two cohorts was positive for anti-Mi-2. In all DM patients (Table 3, left), men appeared to be more common in anti-Mi-2 (+) versus (-) (37% versus 22%) but this was not statistically significant. Among DM features, the shawl sign was more common in anti-Mi-2 (+) (86% versus 61% in anti-Mi-2 (-), *P *< 0.05) but the prevalence of heliotrope rash and the Gottrön sign was similar. However, the prevalence of the shawl sign was similar when anti-Mi-2 (+) versus (-) Mexico City DM patients were compared (85% versus 78%); the difference was due to the low prevalence of the shawl sign in anti-Mi-2 (-) DM patients from Guadalajara (Mexico City versus Guadalajara, 78% versus 40%, *P *< 0.05). Malignancy was found in two cases of PM (a case of cervical cancer from Guadalajara, no MSA; a case of breast cancer from Mexico City, anti-Jo-1) and two cases in anti-Mi-2 (-) DM from Mexico City (a case of breast cancer, anti-SAE; a case of cervical cancer, anti-p155/140). Among 10 anti-p155/140 positive DM patients, only one had a malignancy (cervical cancer), likely related to the generally young age of the cohort, due to genetic and environmental factors or other reasons.

**Table 1:** Prevalence of autoantibodies in Mexican patients with PM/DM

	PM/DM	PM	DM	Adult-onset DM	Juvenile- onset DM
Number	95	29	66	61	5
Age	44.4	48.4	42.8	45.3	18.2
Mean ± SD	± 14.2	± 10.2	± 15.3	± 14.4	± 9.7
Male	33%	41%	29%	28%	40%
Autoantibodies					
Jo-1	4%	7%	3%	3%	0%
Mi-2 (95% CI)	35%	10%^a^ (2.1 to 26.5)	45%^a^ (33.1 to 58.2)	45%	40%
p155/140 (95% CI)	11%	0%^b^ (0.0 to 11.9)	15%^b^ (7.5 to 26.1)	16%	0%
MJ/NXP-2	3%	0%	5%	3%	20%
SAE	2%	0%	3%	3%	0%
SRP	3%	7%	2%	2%	0%
PM-Scl	3%	0%	5%	5%	0%
U1RNP	3%	7%	2%	2%	0%
Negative	36%	69%^c^	21%^c^	20%	40%
Su/Ago2	8%	7%	9%	10%	0%
Ro60	13%	7%	15%	15%	20%
Ro52^d^	21%	16%	22%	24%	0%

^a^*P *< 0.0001; ^b^*P *< 0.05; ^c^*P *< 0.0001 by Fisher exact test; ^d^some samples were not tested due to availability. All were determined by immunoprecipitation except anti-Ro52 determined by ELISA. Anti-EJ, PL-7, PL-12, OJ were not found. DM, dermatomyositis; negative, negative for myositis-specifc/related autoantibodies, anti-Jo-1 to -U1RNP in the table; PM, polymyositis; SAE, small ubiquitin-like modifier activating enzyme; SRP, signal recognition particle.

**Table 2:** Prevalence of myositis-specific/related autoantibodies in adult-onset DM

	Adult-onset DM Mexico City	Adult-onset DM Guadalajara
Number	44	17
Age (mean ± SD)	46.7 ± 14.1^a^	39.9 ± 12.1^a^
Male	32%	18%
Autoantibodies		
Jo-1	4%	0%
Mi-2 (95% CI)	59%^b^ (43.3 to 73.7)	12%^b^ (1.5 to 36.4)
p155/140 (95% CI)	9%^c^ (2.5 to 21.7)	35%^c^ (14.2 to 61.7)
MJ/NXP-2	4%	6%
SAE	4%	0%
SRP	0%	6%
PM-Scl	2%	12%
U1RNP	2%	0%
Negative	16%	29%
Su/Ago2	14%	0%
Ro60	11%	24%
Ro52^d^	21%	29%

^a^*P *= 0.035 by Mann-Whitney; ^b^*P *= 0.0012; ^c^*P *= 0.02, by Fisher exact test; ^d^some samples were not tested due to availability. All were determined by immunoprecipitation except anti-Ro52 determined by ELISA. Anti-EJ, PL-7, PL-12, OJ were not found. DM, dermatomyositis; negative, negative for myositis-specifc/related autoantibodies, anti-Jo-1 to -U1RNP in the table; PM, polymyositis; SAE, small ubiquitin-like modifier activating enzyme; SRP, signal recognition particle.

**Table 3:** Clinical characteristics of DM patients with versus without anti-Mi-2

	All DM Number = 66		Mexico City DM Number = 46		Guadalajara DM Number = 20	
	**Anti-Mi-2** **(+)**	**Anti-Mi-2** **(-)**	**Anti-Mi-2** **(+)**	**Anti-Mi-2** **(-)**	**Anti-Mi-2** **(+)**	**Anti-Mi-2** **(-)**

Number	30	36	27	19	3	17
Age	41.3	44.1	42.0	50.3	34.7	37.2
(mean ± SD)	± 14.2	± 16.3	± 14.7^a^	± 15.1^a, b^	± 4.5	± 15.3^b^
% male	37%	22%	33%	26%	67%	18%
Shawl sign	86%^c^	61%^c^	85%	78%^d^	100%	40%^d^
Heliotrope	74%	79%	71%	89%	100%	69%
Gottrön	78%	74%	79%	78%	67%	69%
Malignancy	0%	6%	0%	11%	0%	0%
Fever	30%	25%	25%	28%	67%	21%
Calcinosis	7%	9%	4%	5%	33%	13%
ILD	7%	9%	4%	17%	50%	0%
Initial CPK IU/L (mean ± SD)	5,369 ± 4,124^e^	2,860 ± 5,183^e^	4,800 ± 3,111^f^	3,502 ± 6,068^f, g^	NA	1,808 ± 3,475^g^
Initial CPK < 200 IU/L	0%^h^	34%^h^	0%^i^	22%^i, j^	NA	55%^j^
Initial CPK > 1,000 IU/L	100%^k^	52%^k^	100%^l^	67%^l, m^	NA	27%^m^
Initial CPK > 5,000 IU/L	54%^n^	14%^n^	52%^o^	17%^o^	NA	9%
Last CPK > 500 IU/L	37%^p^	7%^p^	15%	0%	NA	18%
Last CPK < 200 IU/L	45%	69%	45%	67%	NA	73%

^a^*P *< 0.05; ^b^*P *< 0.005; ^c^*P *< 0.05; ^d^*P *< 0.05; ^e^*P *< 0.0005; ^f^*P *< 0.01; ^g^not significant; ^h^*P *< 0.005; ^i^*P *< 0.05; ^j^*P *= 0.11; ^k^*P *< 0.0001; ^l^*P *< 0.005; ^m^*P *= 0.06; ^n^*P *< 0.005; 15, ^o^*P *< 0.05; ^p^*P *= 0.095. CPK, creatine phosphokinase; DM, dermatomyositis; ILD, interstitial lung disease; NA, not applicable (due to small numbers).

The prevalence of interstitial lung disease (ILD) was low compared with other studies, likely due to the very low prevalence of anti-ARS antibodies that are frequently associated with ILD (2). CPK at the initial visit was significantly higher in anti-Mi-2 (+) versus (-) DM patients (mean 5,369 versus 2,860 IU/L, *P *< 0.0005) (Table 3 and Figure 1B) and this trend was the same when Mexico City DM patients were separately analyzed (4,800 versus 3,502 IU/L, *P *< 0.01). Starting lactate dehydrogenase (LDH) levels were also higher in the anti-Mi-2 (+) (mean ± SD, 1,517.17 ± 805.02) than in the anti-Mi-2 negative group (841.41 ± 605.86) (*P *< 0.005 by Mann-Whitney). None of the patients with anti-Mi-2 (+) had normal (< 200) CPK at the initial visit whereas 34% of the anti-Mi-2 (-) patients (*P *< 0.005) had normal levels, which was also consistent in Mexico City patients (*P *< 0.05). All 30 anti-Mi-2 (+) patients had CPK > 1,000 IU/L at the initial visit compared with only 52% in anti-Mi-2 (-) patients (*P *< 0.0001). CPK > 5,000 IU/L was more common in anti-Mi-2 (+) versus anti-Mi-2 (-) patients (54% versus 14%, P < 0.005). Although CPK levels in anti-Mi-2 (+) patients were very high, they responded well to steroid therapy and CPK levels dropped dramatically in most patients (P < 0.0001) (Figure 1C), similar to the anti-Mi-2 (-) group. At the last visit, the percentage of patients who had CPK > 500 IU/L was 37% in the anti-Mi-2 (+) versus 7% in the anti-Mi-2 (-) group (*P *= 0.095); however, 45% of the former group had normal or near normal CPK levels of < 200 IU/L.

## Discussion

Autoantibodies to Mi-2 were originally defined by double immunodiffusion using calf thymus extract as the antigen and reported as the first specific serologic marker of DM [22]. IP was applied and a target antigen complex was characterized [16], which was later identified as nucleosome remodeling deacetylase complex (NuRD) [23]. Clinically, most of the anti-Mi-2 positive patients are DM [22,24-26]; however, clinical characteristics associated with anti-Mi-2 have not been studied extensively due to limited availability of the immunoassays and relatively low prevalence in PM/DM. The present study, with a total of 33 anti-Mi-2 positive patients confirmed by IP, is the largest cohort in the literature with a detailed clinical analysis of anti-Mi-2 positive patients. Clinical features of anti-Mi-2 positive patients in this study were consistent with previous literature; they were mostly DM with typical skin manifestations, and low prevalence of ILD and malignancy [24]. Initial CPK levels were higher in the anti-Mi-2 positive DM group compared with the negative group (Table 3, Figure 1B). However, they responded well to steroid treatment in general; CPK levels were reduced dramatically and normalized in many cases (Table 3, Figure 1C), consistent with the good prognosis reported in the literature [24]. The high prevalence of anti-Mi-2 (35%) and the low prevalence of anti-synthetase antibodies (4%) in Mexican PM/DM cases was the most striking finding in the present study (Table 1) compared with MSAs in other ethnicities. Anti-Jo-1 which is the most common MSA in the majority of studies [4,19] was found only in 4% of the PM/DM patients (7% in the PM and 3% in the DM). The prevalence of anti-Mi-2 was 35% in PM/DM and was increased to 45% in DM patients (Table 1). Data on the prevalence of MSAs in Mexicans are limited to basically one cohort in Mesoamerican Mestizo from Mexico City and Guadalajara [5,6]. Our data are consistent with the study that reported a high prevalence of anti-Mi-2 and a low prevalence of anti-synthetase antibodies, in Mexican PM/DM [6] (Table 4). A correlation of UV radiation with the development of DM and production of anti-Mi-2 antibodies has been reported [6,27]. The role of sunlight in anti-Mi-2 production through alterations in the expression, subcellular distribution, and/or metabolism of components of the Mi-2 antigens, has been suggested [6]. Supporting this hypothesis, regulation of Mi-2 expression by UV through protein translation and stability has been shown [28]. Although the high prevalence of anti-Mi-2 in Mexican PM/DM was interpreted in association with high UV radiation in the area [6], the roles of genetic versus environmental factors responsible for the production of anti-Mi-2 are not fully understood. Data on MSAs in the Mexican population living in the US to compare with these data are very limited. One study reported 8% prevalence of anti-Mi-2 in Mexican American PM/DM cases in Texas [4], which is not higher than other ethnicities and is lower than that of Mestizo living in Mexico [6]. This may seem to be consistent with the role of environmental factors, in particular UV radiation in anti-Mi-2 antibody production. However, a significant difference in the prevalence of anti-Mi-2 in Mexico City versus Guadalajara, which have comparable UV radiation levels, was observed in two independent studies by Okada *et al. *[6] and in the present study (Table 4). A significant difference in the prevalence of anti-Mi-2 in PM/DM patients in Mexico City versus those in Guadalajara (43% versus 14%, *P *= 0.0088, Table 4) was found in our study. Interestingly, a similar pattern of prevalence of anti-Mi-2 in Mexico City versus Guadalajara PM/DM cases (36% versus 13%, *P *= 0.03 by Fisher's exact test based on our calculation, not shown or discussed in the original study [6]) was also reported in a previous independent study [6]. When data from the two studies were combined (see Table 4, row 'anti-Mi-2 in PM/DM, combined'), the prevalence of anti-Mi-2 in Mexico City versus Guadalajara patients was 41% versus 14% (*P *< 0.0001) with no overlap in 95% CI. Despite relatively small numbers of DM in the present study, no overlap in 95% CI was seen in the prevalence of anti-Mi-2 in Mexico City versus Guadalajara patients (59% versus 15%, *P *= 0.001) (Table 4, row anti-Mi-2 in DM). Very similar data from two independent studies and statistical difference with no overlap of 95% CI support the interpretation that the difference between the two cities/cohorts is real. Interestingly, the percentage of DM in PM/DM in the two locations is similar, approximately 79% in the previous study [6] and 69% to 71% in the present study, indicating that the different prevalence of anti-Mi-2 is not a reflection of a difference in PM:DM ratios. These data suggest that anti-Mi-2 production is affected by undetermined differences in the two Mexico locations of genetic and/or environmental factors other than UV radiation alone. While environmental factors are poorly characterized, significant differences in genetic background of Mexican-Mestizo in the two locations have been reported [29]. Y-chromosome short-tandem repeats-based admixture estimates of African: European: Amerindian in the Mexican-Mestizo population in Mexico City is 4%:46%:50% whereas that in Jalisco County (where Guadalajara is located), is 5%:67%:28%, indicating a higher percentage of Amerindian derived genes in Mexico City and European derived gene dominance in Jalisco County [29]. This heterogeneous genetic background among Mexicans could also be an important issue when interpreting the reported low prevalence of anti-Mi-2 in Mexican-Americans [4] as the admixture of Mexican Americans in Texas [30] appears to be very similar to that of Jalisco County (Guadalajara) [29] that also has a low prevalence of anti-Mi-2 (Table 4). Other environmental factors not characterized in the previous studies may also be important in an unexplained accumulation of patients with a specific MSA to a certain year or geographical location, which does not appear to be due to genetic heterogeneity [10,11].

**Table 4:** Comparison of percentage of DM, prevalence of anti-Mi-2 and genetic origin in the literature

	Mexico City		Guadalajara		Texas
	**Mestizo**	**Mestizo**	**Mestizo**	**Mestizo**	**Mexican American**

	**Okada **[6]	**Present study**	**Okada **[6]	**Present study**	**Arnett **[4]

PM/DM number	36	67 (46 DM)	38	28 (20 DM)	25
% of DM in PM/DM	79%	69%	79%	71%	NA
Anti-Mi-2 in PM/DM (95% CI)	36%^a^ (20.8 to 53.8)	43%^b, c^ (31.2 to 56.0)	13%^a^ (4.4 to 28.1)	14%^b^ (4.0 to 32.7)	8%^c^ (1.0 to 26.0)
Anti-Mi-2 in PM/DM, combined (95% CI)	41%^d, e^ (31.2 to 50.9)		14%^d^ (6.4 to 24.3)		8%^e^ (1.0 to 26.0)
Anti-Mi-2 in DM (95% CI)	NA	59%^f^ (43.2 to 73.0)	NA	15%^f^ (3.2 to 37.9)	NA
Anti-synthetase in PM/DM	4%	6%	0%	0%	20%
UV index [32]	10.5 to 12.5		10.5 to 12.5		6.5 to 8.5
Genetic admixture estimate [29,30]					
African	4%		5%		8%
European	46%		67%		61%
Amerindian	50%		28%		31%

^a^*P *= 0.03; ^b^*P *= 0.0088; ^c^*P *= 0.0012; ^d^*P *< 0.0001; ^e^*P *= 0.0018; ^f^P = 0.001. DM, dermatomyositis; PM, polymyositis

## Conclusions

Both genetic and environmental factors affect the phenotypes of autoimmune diseases; however, there are still unidentified or poorly characterized factors present and sometimes their contribution may not be apparent. The present study emphasizes the importance of careful analysis, considering the heterogeneity of the population that may also be related to genetic and/or environmental factors to understand better the mechanisms of MSA production [31].

## Abbreviations

ARS: aminoacyl tRNA synthetases; CPK: creatine phosphokinase; ELISA: enzyme-linked immunosorbent assay; IgG: immunoglobulin G; ILD: interstitial lung disease; IP: immunoprecipitation; JDM: juvenile-onset dermatomyositis; LDH: lactate dehydrogenase; MSAs: myositis-specific autoantibodies; NuRD: nucleosome remodeling deacetylase complex; PM/DM: polymyositis/dermatomyositis; SAE: small ubiquitin-like modifier activating enzyme; tRNA: transfer RNA.

## Competing interests

The authors declare they have no competing interests.

## Authors' contributions

MHP helped in data collection and writing the manuscript. MS designed the study, performed experiments, analyzed data and wrote the manuscript. BTMM helped in data collection and writing the manuscript. RVR helped in recruitment of patients, experimental techniques and writing the manuscript. LJJ, MAS, CCG, LAO, OVL, MSP, RMPP, LGL and JIGN helped in recruiting patients, collecting clinical information and writing the manuscript. HURS helped in designing the study and writing the manuscript. JYFC, SJR and EKLC helped in obtaining and analyzing data and writing the manuscript. MVDM designed the study, collected data and wrote the manuscript. All authors have read and approved the final manuscript.
